# High-Intensity Endurance Training Results in Faster Vessel-Specific Rate of Vasorelaxation in Type 1 Diabetic Rats

**DOI:** 10.1371/journal.pone.0059678

**Published:** 2013-03-19

**Authors:** Juan M. Murias, Adwitia Dey, Oscar A. Campos, Mehrbod Estaki, Katharine E. Hall, Christopher W. J. Melling, Earl G. Noble

**Affiliations:** 1 School of Kinesiology, Western University, London, Ontario, Canada; 2 School of Health Studies, Western University, London, Ontario, Canada; 3 Lawson Health Research Institute, Western University, London, Ontario, Canada; The Chinese University of Hong Kong, Hong Kong

## Abstract

This study examined the effects of 6 weeks of moderate- (MD) and high-intensity endurance training (HD) and resistance training (RD) on the vasorelaxation responsiveness of the aorta, iliac, and femoral vessels in type 1 diabetic (D) rats. Vasorelaxation to acetylcholine was modeled as a mono-exponential function. A potential mediator of vasorelaxation, endothelial nitric oxide synthase (e-NOS) was determined by Western blots. Vessel lumen-to-wall ratios were calculated from H&E stains. The vasorelaxation time-constant (τ) (s) was smaller in control (C) (7.2±3.7) compared to D (9.1±4.4) and it was smaller in HD (5.4±1.5) compared to C, D, RD (8.3±3.7) and MD (8.7±3.8) (p<0.05). The rate of vasorelaxation (%·s^−1^) was larger in HD (2.7±1.2) compared to C (2.0±1.2), D (2.0±1.5), RD (2.0±1.0), and MD (2.0±1.2) (p<0.05). τ vasorelaxation was smaller in the femoral (6.9±3.7) and iliac (6.9±4.7) than the aorta (9.0±5.0) (p<0.05). The rate of vasorelaxation was progressively larger from the femoral (3.1±1.4) to the iliac (2.0±0.9) and to the aorta (1.3±0.5) (p<0.05). e-NOS content (% of positive control) was greater in HD (104±90) compared to C (71±64), D (85±65), RD (69±43), and MD (76±44) (p<0.05). e-NOS normalized to lumen-to-wall ratio (%·mm^−1^) was larger in the femoral (11.7±11.1) compared to the aorta (3.2±1.9) (p<0.05). Although vasorelaxation responses were vessel-specific, high-intensity endurance training was the most effective exercise modality in restoring the diabetes-related loss of vascular responsiveness. Changes in the vasoresponsiveness seem to be endothelium-dependent as evidenced by the greater e-NOS content in HD and the greater normalized e-NOS content in the smaller vessels.

## Introduction

Diabetes mellitus is related to vascular problems such as endothelial dysfunction [1]–[3] and early development of microvascular disease [4]–[6]. Production of nitric oxide (NO) by the endothelial cells results in the release of this vasoactive gas into the smooth muscle, which results in endothelium-dependent vasorelaxation. Thus, the endothelium plays a key role in controlling vascular tone and function [7], [8]. However, hyperglycemia and diabetes lead to a reduction in NO production and activity [1], which might affect vascular function and thereby provision of oxygen and other nutrients to the organs and negatively affect cellular energetics and homeostasis [9]. Recently, we have demonstrated that the dynamic adjustment of the endothelium-dependent vasorelaxation response was impaired in type 1 diabetic compared to control rats in different vessels throughout the vascular tree [10] Importantly, this reduced responsiveness of the vessels was observed using a model in which diabetic rats were supplemented with exogenous insulin to mimic a “poorly controlled” diabetic [11].

Although the positive effects of exercise training on the vasculature (and more specifically endothelium dependent vasodilation) have been demonstrated [12]–[17], it is presently unclear what the ideal exercise type and dose are to produce favourable changes in the general and diabetic populations [16]. For instance, it has been reported that high-intensity endurance exercise is most beneficial in preventing type II diabetes and preserving cardiovascular function compared to low-intensity endurance exercise [18]. Also, higher intensity endurance exercise is likely to remodel the vascular system to a greater degree than lower intensity activities [19] leading to beneficial endothelial and vascular adaptations [20]. However, acetylcholine (ACh)-mediated forearm vasodilation in response to endurance exercise training has been shown to improve in those performing at moderate (50% of maximal oxygen uptake (VO_2max_)) but not at low (25% VO_2max_) or high (75% VO_2max_) intensities [13]. Additionally, resistance training has been shown to be beneficial for vascular remodelling of conduit and resistance arteries [21]–[23] but to have no positive effects in measures of arterial compliance [24], [25].

Using animal models, changes in vascular responsiveness to different types of interventions are often evaluated as differences in the absolute and percent dose-response vasorelaxation to vasoactive substances [4], [26]–[28]. However, changes in vascular responsiveness occur not only in the amplitude domain of the response but also in its rate of adjustment. As such, we have recently reported changes in the rate of adjustment of the vasorelaxation response to a given dose of ACh [10]. Using this analysis, we have described the adjustments in the percent vasorelaxation (amplitude) as well as in the dynamic component of the response. In those studies, we also showed that the vasorelaxation response was vessel-specific. Therefore, changes in vascular responsiveness to exercise training interventions should consider both, the percent and the dynamic component of the adjustment, as well as different vessel types that might show dissimilar responses depending on their location and/or function.

Thus, the goal of this study was to evaluate the effects of moderate- and high-intensity endurance training and resistance training on the responsiveness of different vessels throughout the vascular tree (aorta, iliac, and femoral) in type 1 diabetic rats. We hypothesized that: 1) High-intensity endurance training would be most beneficial in improving vascular responsiveness; 2) Changes in both the amplitude and the dynamic adjustment of response would be vessel-specific.

## Methods

### Ethics Statement

This study was approved by the University of Western Ontario Council on Animal Care and was performed in accordance with the guidelines of the Canadian Council on Animal Care.

### Animal Characteristics

Fifty male 8 week-old Sprague Dawley rats were obtained from Charles River Laboratories and housed in pairs in standard rat cages with a 12∶12-hour light-dark cycle in a temperature (20±1°C) and relative humidity (50%) controlled environment. Food and water were provided *ad libitum*. All efforts were made to minimize suffering.

### Experimental Protocol

Rats were randomly assigned to a non-diabetic sedentary control (C; n = 10), sedentary diabetic (D; n = 10) resistance training diabetic (RD; n = 10), and moderate- (MD; n = 10) or high-intensity (HD; n = 10) endurance training diabetic groups. Type 1 diabetes mellitus was induced by giving 20 mg·kg^−1^ of streptozotocin (STZ) via intraperitoneal (I.P.) injection on 5 consecutive days. This approach was selected because it mimics the development of Type I diabetes more closely than a single dose injection of STZ [29]. Diabetes was confirmed when two blood glucose concentrations greater than18 mmol·L^−1^ were measured on consecutive days. If diabetes was not confirmed after five injections, the animals were given subsequent 20 mg·kg^−1^ STZ-I.P. injections until two readings of 18 mmol·L^−1^ were obtained. Once diabetes was confirmed, 1 insulin pellet (LinShin, LinPlant, LinShin Canada Inc., Toronto, ON) was implanted subcutaneously above the abdomen. Insulin pellet dosages were monitored and adjusted to obtain daily non-fasting blood glucose concentrations in the range of 9–15 mmol·L^−1^ to mimic poorly controlled type 1 diabetic patients using a constant dose of insulin and to mitigate the dramatic weight loss and significant organ damage in STZ alone animals [29], [30]. Animals were maintained as controls or exercise trained for an additional six weeks prior to sacrifice.

### Exercise Training Protocol

Prior to the start of the exercise-training program, rats were familiarized with the exercise equipment over five occasions so that they would become comfortable with their surroundings, while steadily increasing their ability to perform the exercise program. For the next five weeks, HD exercised 5 days per week for 1 hour per day on a rodent motor-driven treadmill at a 6 degree slope at a speed of 27 m·min^−1^. MD had a similar regime but the running speed was set at 15 m·min^−1^. These exercise intensities were chosen to represent ∼70–80 and ∼50–60% of VO_2max_ for HD and MD, respectively [31]. Continuous running during the aerobic exercise sessions was encouraged by small blasts of compressed air. RD rats were required to climb a ladder while wearing a weighted bag secured to the proximal portion of their tail. The ladder was 1.1 m tall on an 80 degree incline and with 2 cm spacing between rungs. This protocol describes an animal model of resistance exercise that closely resembles the exercise parameters and physiological adaptations observed in humans who participate in resistance training [32]. The first week of resistance training served as familiarization and consisted of 10 ladder climbs with progressive increases in weight up to 35% of body mass. After each climb, rats were allowed a 2 minute rest in a 20 cm^3^ dark box atop the ladder. Following familiarization, RD rats underwent high intensity progressive resistance training 5 days a week for 5 weeks. The resistance training protocol was adapted from Hornerberger and Farrar [32] as follows: Pre-training maximal carrying capacity was determined by initially loading rats with 75% of their body weight and then adding 30 g per climb until they were unable to successfully climb the ladder. For the first 4 ladder climbs, rats were loaded with 50%, 75%, 90% and 100% of their pre-training maximal carrying capacity. Subsequently, rats continued to climb at 100% maximal carrying capacity until exhaustion (unwillingness to climb despite tactile stimulation on their haunches). Every 3 days, a new maximal carrying capacity was determined. The load corresponding to 100% of the rat’s maximal carrying capacity was 429±57 g at the start of the resistance training program and 608±129 g at the end of the program. These values corresponded to 103±15% and 136±31% of the rat’s body mass at the start and the end of the training intervention, respectively.

### Vessels Collection

Rats were anaesthetized via an I.P. injection of 65 mg·kg^−1^ pentobarbital sodium and were sacrificed via heart excision 18 hours after the last bout of exercise. The aorta, iliac, and femoral arteries were rapidly excised and placed into ice-cold modified Krebs-Henseleit buffer (118.1 mM NaCl, 4.7 mM KCl, 1.5 mM CaCl_2_, 1.2 mM KH_2_PO_4_, 1.2 mM MgSO_4_, 11.1 mM D-glucose, 25 mM NaHCO_3_, pH 7.4). The vessels were then carefully cleaned of connective and adipose tissue. A portion of the abdominal aorta, iliac, and femoral artery were then divided into ∼2 mm long rings and, after removal of luminal blood clots, vessel rings were used for *in vitro* isometric tension measurements.

### In vitro Isometric Tension Analysis

Each vessel ring was mounted onto a GlobalTown Microtech EZ-bath system (GobalTown Microtech Inc., Sarasota, FL) and placed in 5 ml organ baths containing modified Krebs–Henseleit buffer (37°C) that was constantly aerated with 95% O_2_ and 5% CO_2_. Initial ring tension was manually adjusted to ∼2 g in the aorta, ∼1.5 g in the iliac, and ∼1.0 g in the femoral artery. These values were the results of pilot testing in our laboratory to determine optimal baseline tensions for each vessel section, as previously reported [10]. Rings were allowed to equilibrate at these tensions for ∼40 minutes. Fresh buffer (5 ml) was added to organ baths at the end of the equilibration period. Isometric contractions and relaxations were continuously measured using PowerLab (ML856 26T; ADInstruments, Colorado Springs, CO). Data were recorded using LabChart v7.0 (ADInstruments, Colorado Springs, CO) at a sampling rate of 1000 Hz. The vessels were pre-constricted with 10^−5^ M phenylephrine (PE). When a steady-state level of constriction was observed, vasorelaxation of the vessels to a single dose of 10^−4^ M ACh was measured. Following these experiments, vessels were exposed to a single dose of 10^−5^ M of the NOS inhibitor N^G^-nitro-L-arginine methyl ester (L-NAME). Vasorelaxation responses to 10^−4^ M ACh and to 10^−4^ M sodium nitroprusside (SNP) were assessed in the presence of L-NAME in the organ bath. Each organ bath was washed between each condition by flushing the organ bath and adding fresh buffer 3 times every 5 min.

### Data Analysis

The on-transient vasorelaxation responses were modelled using this equation:
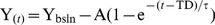
(1)where Y_(*t*)_ is the tension (g) at any given time (*t*); Y_Bsln_ is the steady state baseline value of Y before a decrease in the tension as a consequence of the vasorelaxation; A is the amplitude of the decrease in Y below Y_Bsln_; τ (time constant of the response) is the time required to attain 63% of the steady-state amplitude; and TD is the mathematically generated time delay through which the exponential model is projected to intersect Y_Bsln_. Vasorelaxation responses were modeled to ∼2 min because the relaxant effects of ACh are known to be transient in nature due to the chemical instability of endothelium-derived relaxing factors [33]. The model parameters were estimated by least-squares nonlinear regression (Origin, OriginLab Corp., Northampton, MA) in which the best fit was defined by minimization of the residual sum of squares and minimal variation of residuals around the Y-axis (Y = 0). The 95% confidence interval (CI_95_) for the estimated time constant was determined after preliminary fit of the data with Y_Bsln_, A, and TD constrained to the best-fit values and the τ allowed to vary. The calculated time delay for the vasorelaxation response (CTD) was estimated using second-by-second data and represented the time, after ACh infusion, at which the signal initiated a systematic decrease from its steady-state constriction value. The time-to-steady-state was calculated as the CTD +4τ (with 4τ being ∼98% of the total adjustment). Baseline constriction values were computed as the mean value in the 30 s prior to a transition.

### Western Blotting

Subsequent to mincing, vessels were homogenized in 100 µL of homogenizing buffer (25 mM Tris, 137 mM NaCl, 1% Triton x-100 (pH 7.4–7.5)) that included a cocktail of protease and phosphatase inhibitors (Sigma P8340, P0044, and P5726) using a glass dounce homogenizer and then transferred into 1.5 mL microcentrifuge tubes. Samples were kept on ice for the entire protocol. The microcentrifuge tubes were later centrifuged at 14,000 g for 10 minutes at 4°C. Supernatants were removed and placed into new microcentrifuge tubes for storage at −70°C. Sample protein content was determined using a Bradford assay and 30–50 µL of protein homogenate was loaded into each well such that each vessel type had the same amount of total protein loaded. A human endothelial cell lysate (BD 611450) was used as the positive control. Electrophoresis was performed and proteins were separated by running through a 6% polyacrylamide gel for ∼1 h at 100 V. Proteins were electrophoretically transferred to a nitrocellulose membrane for 45 min at 100 V. Membranes were blocked with 5% non-fat dry milk (BioRad 170–6404) in Tween-Tris buffered saline (TTBS; 10 mM Tris, 100 mM NaCl, 0.1% Tween-20, pH 7.5) for 1 hour at room temperature. Membranes were washed in TTBS (3×10 min). Membranes were first incubated with primary antibody specific for eNOS (mouse monoclonal anti-eNOS IgG, BD 610297, 1∶2000) overnight at 4°C. The next day, membranes were incubated with horseradish peroxidase-conjugated secondary antibody specific for mouse IgG (goat anti-mouse IgG-HRP conjugate, BioRad 170–6516, 1∶5000). Protein bands were visualized and captured on digital camera using a BioRad Chemidoc XRS imager and optical density was quantified using BioRad Quantity One software. Optical density was normalized to the appropriate standards.

### Lumen-to-wall Measurements

A portion of the abdominal aorta, iliac, and femoral arteries was isolated, cleaned, and perpendicularly mounted on cork embedded in OCT compound (Thermo Fisher Scientific Inc., Waltham, MA) before the samples were snap frozen in isopentane that had been pre-cooled in liquid nitrogen, and cryopreserved at −80°C. Frozen tissues were transversely sectioned into 10 µm thick slices on a Leica 1770 cryostat (Leica Microsystems, Wetzlar, Germany) at −20°C and mounted on positively charged VWR Suprafrost plus microscope slides (VWR International, Radnor, PA). The slides were stored at −20°C for short term storage and thawed for 30 minutes prior to staining. Routine hematoxylin and eosin staining was carried out on all the slides (two serial sections per animal). The vessel lumen to wall ratio was calculated using ImageJ v.1.47 software whereby the wall is the width of the smooth muscle intima. The width of the smooth muscle intima was calculated by subtracting the lumen diameter from the total diameter of the vessel. The difference was then halved to obtain the width of the smooth muscle intima. The lumen to wall ratio was defined as lumen diameter to the calculated width of the smooth muscle intima.

### Statistics

Data are presented as means ± SD. A two-way analysis of variance (ANOVA) was used to determine statistical significance for the dependent variables. The ANOVA model was described as G_5_×V_3_ such that groups (G; C, D, RD, MD, and HD) is crossed with vessels (V; aorta, iliac and femoral). A Tukey post-hoc analysis was used when significant differences were found for the main effects of each dependent variable. The ANOVA was analyzed by SPSS Version 20.0, (SPSS Inc., Chicago, IL). Statistical significance was declared when p<0.05.

## Results

Basal glucose concentration before the rats were assigned to each group was 5.62±0.68 mmol·L^−1^. Fasting blood glucose concentration was kept within 9 and 15 mmol·L^−1^ throughout the experiments. The amount of insulin supplementation required to maintain that glucose concentration (as presented elsewhere, Hall et al., in revision for *“Metabolism: clinical and experimental”*) was significantly lower (p<0.05) in HD (3.9±0.2 IU·kg·day^−1^) compared to D (5.6±0.4 IU·kg·day^−1^), RD (5.6±0.4 IU·kg·day^−1^), and MD (4.6±0.7 IU·kg·day^−1^) and in MD compared to D and RD.

**Figure 1:**
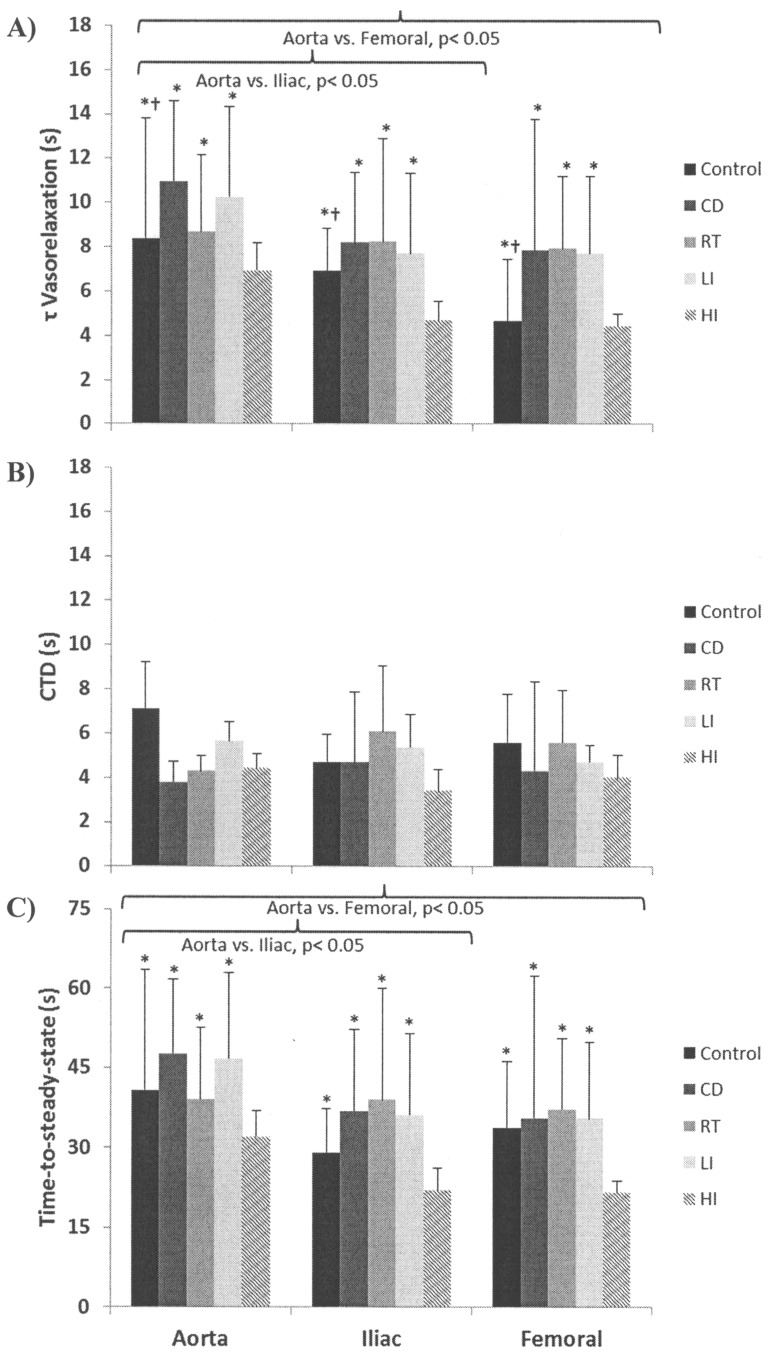
Time constant (τ) (A), calculated time delay (CTD) (B), and time-to-steady-state (C), responses for each vessel and group. Values are means ± (SD). D, diabetic animals; RD, resistance trained diabetic animals; MD, moderate intensity endurance trained diabetic animals; HD, high intensity endurance trained animals; *significantly different from HD (p<0.05); ^†^significantly different from D (p<0.05).

**Figure 2:**
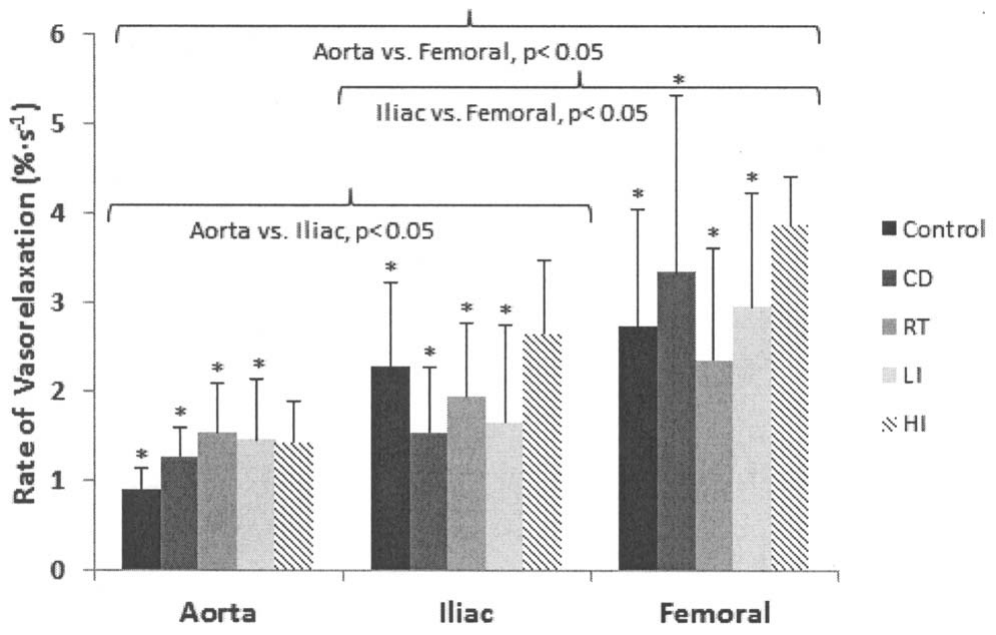
Rate of vasorelaxation for each and group. Values are means ± (SD). D, diabetic animals; RD, resistance trained diabetic animals; MD, moderate intensity endurance trained diabetic animals; HD, high intensity endurance trained animals; *significantly different from HD (p<0.05).

Kinetics parameters, the percent relaxation, and the rate of vasorelaxation for each vessel in each condition are presented in Table 1 and in Figure 1 (A–C) and Figure 2. The overall τ was smaller in C (7.2±3.7 s) compared to D (9.1±4.4 s) and it was also smaller in HD (5.4±1.5 s) compared to C, D, RD (8.3±3.7 s) and MD (8.7±3.8 s) (p<0.05) (Figure 1 A). There was no significant main effect for the exercise training interventions for the CTD (p>0.05) (Figure 1B). The time-to-steady-state of the vasorelaxation response was faster in HD (25.3±6.1 s) compared to C (34.5±16.0 s), D (40.4±19.4 s), RD (38.5±15.8 s), and MD (40.0±15.7 s) (p<0.05) (Figure 1 C). Exercise training resulted in no changes for the % vasorelaxation response (C, 59±25%; D, 64±21%; RD, 64±16%; MD, 67±24%; HD, 62±20%) (p>0.05). The rate of vasorelaxation was significantly larger in HD (2.7±1.2%·s^−1^) compared to C (2.0±1.2%·s^−1^), D (2.0±1.5%·s^−1^), RD (2.0±1.0%·s^−1^), and MD (2.0±1.2%·s^−1^) (p<0.05) (Figure 2). There was a trend to an exercise training by vessel-type interaction (p = 0.06) suggesting that the rate of vasorelaxation in the aorta was larger in HD compared to C and D but not RD and MD.

**Table 1:** Vasorelaxation kinetics parameters, percent vasorelaxation, and rate of vasorelaxation in the aorta, iliac, and femoral in control (C), diabetic (D), resistance training diabetic (RD), moderate (MD)- and high (HD)-intensity endurance training diabetic groups.

	Aorta					Iliac					Femoral				
	C (n = 10)	D (n = 10)	RD (n = 10)	MD(n = 9)	HD (n = 10)	C (n = 10)	D (n = 9)	RD (n = 10)	MD(n = 8)	HD (n = 10)	C (n = 10)	D (n = 9)	RD (n = 10)	MD (n = 7)	HD (n = 10)
τ Vasorelaxation (s) #, §	8.4* † (5.4)	11.0*(3.6)	8.7* (3.5)	10.3* (4.1)	6.9 (1.2)	6.1* † (1.9)	8.2* (3.2)	8.2* (4.7)	7.7* (3.6)	4.7 (0.9)	7.0* † (2.7)	7.8* (5.9)	7.9* (3.3)	7.7* (3.5)	4.4 (0.5)
CTD Vasorelaxation (s)	7.1 (2.1)	3.8 (0.9)	4.3 (0.7)	5.7 (0.9)	4.4 (0.7)	4.7 (1.3)	4.2 (3.2)	6.1 (3.0)	5.4 (1.5)	3.4 (1.0)	5.6 (2.2)	4.3 (4.0)	5.6 (2.4)	4.7 (0.8)	4.0 (1.1)
Time-to-steady-state (s) #, §	40.7*(22.9)	47.7*(14.0)	39.1*(13.6)	46.7*(16.3)	32.1 (4.8)	29.0* (8.2)	37.0*(15.2)	39.0*(21.0)	36.2*(15.4)	22.1 (4.1)	33.7*(12.6)	35.7* (26.7)	37.3* (13.3)	35.5* (14.4)	21.8 (2.2)
Vasorelaxation (%) #, ‡	33.0 (7.2)	59.9(18.1)	55.1 (12.5)	64.1(23.7)	44.8 (10.6)	62.5 (21.2)	50.2 (16.7)	62.2 (6.9)	48.5 (16.2)	56.4 (11.0)	80.0 (13.3)	81.0 (17.7)	75.1 (18.7)	91.0 (9.7)	84.2 (9.4)
Rate of vasorelaxation (%·s^−1^) #, §, ‡	0.9* (0.2)	1.3* (0.3)	1.5* (0.6)	1.5* (0.7)	1.4 (0.4)	2.3* (1.0)	1.5* (0.7)	1.9* (0.8)	1.7* (1.1)	2.7 (0.8)	2.7* (1.3)	3.3* (2.0)	2.4* (1.3)	3.0* (1.3)	3.9 (0.5)
Vasorelaxation ACh+L-NAME (%)	−1 (3)	1 (3)	−2 (2)	−2 (3)	−1 (2)	−3 (4)	−1 (5)	−3 (6)	−3 (6)	−3 (6)	5 (14)	5 (10)	2 (9)	1 (7)	3 (7)
Vasorelaxation SNP (%)	92 (7)	98 (2)	96 (2)	95 (5)	95 (3)	93 (4)	97 (3)	95 (4)	94 (3)	92 (3)	99 (4)	101 (6)	99 (4)	99 (1)	97 (7)

Values are means ± (SD). C, control animals; D, diabetic animals; RD, resistance trained diabetic animals; MD, moderate intensity endurance trained diabetic animals; HD, high intensity endurance trained animals; n, number of animals; τ, time constant for the vasorelaxation response; CTD, calculated time delay for the vasorelaxation response; Time-to-steady-state, CTD +4 τ; Rate of vasorelaxation, percent vasorelaxation/time-to-steady-state; L-NAME, N^G^-nitro-L-arginine methyl ester; SNP, sodium nitroprusside;

* significantly different from HD (p<0.05);

† significantly different from D (p<0.05);

# femoral significantly different from aorta (p<0.05);

‡ femoral significantly different from iliac (p<0.05).

§ iliac significantly different from aorta (p<0.05).

Kinetics analysis by vessel type showed a smaller τ and shorter time-to-steady-state for vasorelaxation responses in the femoral (6.9±3.7 s and 32.6±16.0 s, respectively) and iliac (6.9±4.7 s and 32.4±14.9 s, respectively) compared to the aorta (9.0±5.0 s and 41.1±15.8 s, respectively) (p<0.05) (Figure 1 A and C). The CTD of the response was similar in all vessels (p>0.05) (Figure 1 B). The % vasorelaxation was larger in the femoral (82±15%) compared to the iliac (56±16%) and the aorta (51±19%) (p<0.05). The rate of vasorelaxation was progressively larger from the femoral (3.1±1.4%·s^−1^) to the iliac (2.0±0.9%·s^−1^) and to the aorta (1.3±0.5%·s^−1^) (p<0.05) (Figure 2).

Vasorelaxation response to ACh were abolished in the presence of L-NAME (C, 0±8%; D, 2±7%; RD, −1±7%; MD, −1±5%; HD, 0±5%). Addition of SNP to the organ bath restored the vasorelaxation response of the vessels (C, 95±6%; D, 99±4%; RD, 97±4%; MD, 96±4%; HD, 95±5%).

**Figure 3:**
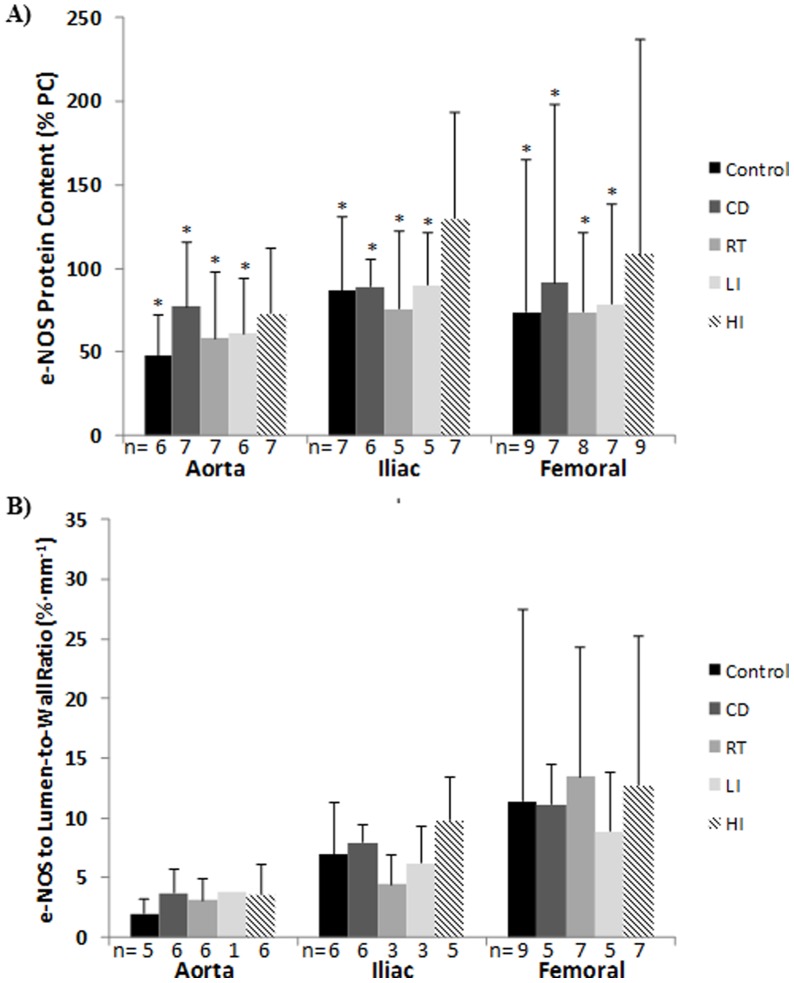
e-NOS protein content and e-NOS content normalized to the lumen-to-wall-ratio for each vessel and group. Values are means ± (SD). D, diabetic animals; RD, resistance trained diabetic animals; MD, moderate intensity endurance trained diabetic animals; HD, high intensity endurance trained animals; n, number of animals; *significantly different from HD (p<0.05).

Figure 3 A–C displays the e-NOS protein content and e-NOS content normalized to the lumen-to-wall ratio for each vessel in each condition. Exercise training resulted in a significantly larger e-NOS content in HD (104±90% positive control) compared to C (71±64%), D (85±65%), RD (69±43%), and MD (76±44%) (p<0.05) (Figure 3 A). No significant main effects were observed in the e-NOS content normalized to the lumen-to-wall ratio. e-NOS protein content was similar in the femoral (85±89% positive control) and iliac (96±46%) compared to the aorta (64±35%) (p = 0.13 and 0.07, respectively) (Figure 3 A). The e-NOS content normalized to the lumen-to-wall ratio was significantly larger in the femoral (11.7±11.1%·mm^−1^) compared to the aorta (3.2±1.9%·mm^−1^) (p<0.05) but not in the femoral compared to the iliac (7.4±3.4%·mm^−1^; p = 0.05) and in the iliac compared to the aorta (p = 0.13) (Figure 3 B).

## Discussion

This study investigated the effects of moderate- and high-intensity endurance training and resistance training on the vasorelaxation responses of different vessels in type 1 diabetic rats. The main findings were that: 1) Diabetes negatively affected the responsiveness of the vessels as shown by a smaller τ vasorelaxation and a trend to a longer time-to-steady-state of the response; 2) high-intensity endurance training reversed the negative effects of diabetes so that the responsiveness of the vessels was restored; 3) the vasorelaxation response was vessel-specific with the aorta displaying the slowest adjustment and the femoral and iliac showing the fastest responsiveness and the femoral presenting the greatest rate of vasorelaxation.

The present data showing a slower rate of vasorelaxation in type 1 diabetic compared to control rats indicate detrimental effects of diabetes on vascular function. Importantly, this study used exogenous insulin supplementation to mimic a “poorly controlled” type 1 diabetic condition [11] rather than using an extremely severe type 1 diabetic model in which insulin production is almost abolished and blood glucose increases to levels beyond what is normally observed in type 1 diabetic humans. Even under this “controlled” condition, diabetic rats showed poorer responsiveness of the vessels after only ∼7–8 weeks from the onset of the disease. These results are in agreement with previous findings showing that diabetes has been associated with vascular problems such as endothelial dysfunction [1]–[3], microvascular disease [4]–[6] and reduced vasoresponsiveness [10]. Remarkably, 6 weeks of high-intensity endurance training resulted in these detrimental effects of diabetes being abolished, as shown by a smaller τ and a shorter time-to-steady-state as well as a greater rate of vasorelaxation in the high-intensity trained rats compared to the other groups. Although previous studies in healthy populations indicated that higher intensities of endurance exercise could be hazardous to human vessels [34] and result in decreased endothelium-dependent vasodilation [13], [35], a meta-analysis showed that higher intensities of exercise might elicit the most beneficial cardiovascular results in diabetic populations [36]. The present data are in line with this later observation. Nevertheless, it is important to note that, although high-intensity endurance training resulted in the fastest vasorelaxation response compared to any of the other groups, the τ vasorelaxation in the MD and RD groups was not significantly different from that observed in the control group. This might indicate the onset of a positive adaptation in response to exercise training in these two groups as well. The reasons for the lack of improvement in the MD compared to the HD group cannot be discerned from this study. However, it could be speculated that lower intensities of endurance training might require a longer duration of training to produce positive vascular adaptations. Another factor to consider is that it might be that the total energy expenditure and not the training intensity per se that modulates some of the adaptations observed in response to exercise training programs [37]. For instance, both the MD and HD groups had the same volume of training but the higher intensity in HD would result in larger energy expenditure in this group compared to MD. Nevertheless, it has been shown that exercise training interventions of very short duration and maximal intensity resulted in similar training adaptations compared to those of longer duration and lower intensity in both health and diseased populations, despite a much lower total amount of work [38].

Another important outcome from this investigation was the confirmation that the rate of adjustment of the vasorelaxation response is vessel-specific such that the femoral and the iliac arteries showed a smaller τ and a shorter time-to-steady-state compared to the aorta, the percent vasorelaxation was the largest in the femoral, and the rate of vasorelaxation was progressively slower from the femoral to the aorta. Using this type of analysis, we have recently shown similar results in the femoral, iliac, and aorta arteries [10] and a markedly slower vascular responsiveness in the carotid artery [39]. As we previously suggested, it is likely that the fastest rate of adjustment observed in the femoral and the iliac is related to the close proximity of these vessels to the muscles that are active during locomotion. In agreement with this idea, it has been proposed that endothelium dependent increases in vasoresponsiveness and blood flow after endurance training were more predominant in the area of muscle tissue with the greatest increase in fiber activity as a consequence of the exercise intervention so that vasorelaxation to ACh and e-NOS protein expression were increased in those areas [40]. In support of the idea that the spatial distribution and location of the vessels plays a role in the vasorelaxation response, other studies have shown that the responsiveness of the arterioles were as fast or even faster than what we observed in larger conduit arteries, and that this responsiveness becomes progressively faster in more distal arterioles (τ∼6 s in first order arterioles and τ∼3 s in third order arterioles) [9], [41]. These results emphasize the importance of describing not only the changes that occur in the amplitude domain of the response but also the dynamic adjustment of the vasorelaxation response in different vessels throughout the vasculature. Although the majority of previous studies using similar vessel myography techniques have used the aorta in their preparations (likely due to its ease of use), this approach might neglect important information from being obtained since the aorta may relax only marginally compared to more distal arteries [42].

What mechanisms might explain this exercise-intensity and vessel-specific improvement in vasorelaxation? The lack of vasorelaxation in response to ACh in the presence of L-NAME, together with the preservation of the vasorelaxation response of the smooth muscle to SNP, confirm the findings from our [10] and other [28], [43], [44] laboratories that endothelium dependent mechanisms are controlling the vasorelaxation responses to ACh. Although prostaglandins and other endothelium-derived relaxing factors might play a role in the vasorelaxation response, it is accepted that NO is the main constituent of this response and that other components might contribute marginally to the exercise-induced changes in vasorelaxation [13]. In this study, differences in e-NOS protein content were evaluated for each exercise modality in each vessel. Our results showed that high-intensity endurance training resulted in the largest e-NOS content of all groups. A larger increase in shear stress in HD, which has been shown to stimulate the activity of NO synthase and the production of NO [45], might explain these results. This is in accordance to Matsumoto et al. [46], who reported that production of NO increases with higher exercise intensities. In opposition to these results, Goto et al. [13] demonstrated that, in young healthy humans, higher intensities of exercise training (∼75% VO_2max_) had no beneficial effects in the endothelium-dependent forearm vasorelaxation response. They speculated that the larger oxidative stress induced by the higher intensity of endurance exercise could have increased oxidative stress to the point of counteracting the potential benefits of an intensity-related increase in NO production. In the presence of diabetes, oxidative stress is already more prominent and NO production is scavenged due to the increased production of superoxide [47]–[49]. Under such a scenario, high-intensity training is perhaps the most effective way of up-regulating NO production and thereby improving vascular responsiveness.

Another factor that could have mediated changes in the vasorelaxation response could be the amount of insulin interacting with the vasculature. In this regard, insulin-mediated changes in vascular responses have been previously demonstrated [50]. As such, it could be expected that the condition that resulted in lower dose of insulin supplementation would likely show a reduction in vasoresponsiveness. However, this was not the case. Thus, the potential effect of blood glucose and insulin concentration on the vasorelaxation response of the vessels examined in the present study seems negligible.

The vessel-specific differences in vasorelaxation response can also be justified by NO production related mechanisms. Albeit not significant, there was a trend towards larger e-NOS protein content in the femoral and iliac (the most responsive vessels) compared to the aorta. Additionally, the e-NOS content normalized to the lumen-to-wall ratio showed a main effect suggesting progressively larger e-NOS content relative to the vessel size from the aorta to the femoral. The use of the lumen-to-wall ratio for normalization of the e-NOS protein content was justified as the diameter of the lumen would be an indirect indicator of the number of endothelial cells available for NO synthesis and the diameter of the wall would indicate the amount of smooth muscle that has to be relaxed upon NO release. Unfortunately, due to limited tissue quantity, the “loss” of some vessels during western blotting plus the “loss” of others vessels during the immunohistochemistry analysis resulted in a small and variable total number of vessels used for this analysis (Figure 3 B). Consequently, the statistical power was reduced, particularly for the analysis by exercise modality (statistics not reported). Notwithstanding this limitation, the observation that smaller and more responsive vessels (with fewer endothelial cells for NO production via e-NOS) have larger e-NOS protein content in relation to the amount of smooth muscle to be relaxed is a novel and important one. Previous studies have shown regional differences in the lumen-to-wall ratio so that smaller arteries, despite the significantly smaller absolute wall thickness, have a relatively larger wall thickness and wall in relation to the lumen area [51]. In that study, a significant correlation between the wall-to-lumen ratio and flow-mediated dilation was observed. The authors suggested that the greater flow-mediated dilation observed in the smaller arteries could be explained, at least in part, by the larger wall-to-lumen ratios. An additional explanation to this response is that the e-NOS protein content, at least in the present data, tends to be larger in the smaller vessels and it is certainly larger when normalized to the lumen-to-wall ratio. As such, the e-NOS concentration is greater in the smaller vessels, which likely leads to a faster vasorelaxation response in our study. However, it should be noted that although the vessels that are more directly affected by the exercise training intervention might be expected to display the most noticeable changes, the positive effects of exercise training in different vessels might be related not only to the benefits in vasodilation that are more specific to the regions were blood flow was actually increased, but also to the general positive effects in vascular health associated with exercise training [52] so that an overall improvement in different vessels through the vasculature should not be unexpected.

In conclusion, this study showed that high-intensity endurance training was the most effective intervention to restore the loss of vascular responsiveness observed in the type 1 diabetic group. Additionally, this investigation confirmed that the dynamic adjustment of the vasorelaxation response is vessel-specific. Endothelium-dependent mechanisms upon production of NO seem to mediate the changes in the responsiveness of the vessels as evidenced by the larger e-NOS protein content in the high-intensity training group and also the larger e-NOS content normalized to the lumen-to-wall ratio in the smaller vessels. These data reinforce the importance of the specificity of the training stimulus to reach the expected benefits and also the relevance of examining vascular responses in different vessels throughout the vascular tree.
